# Germ Tube Mediated Invasion of *Batrachochytrium dendrobatidis* in Amphibian Skin Is Host Dependent

**DOI:** 10.1371/journal.pone.0041481

**Published:** 2012-07-20

**Authors:** Pascale Van Rooij, An Martel, Katharina D'Herde, Melanie Brutyn, Siska Croubels, Richard Ducatelle, Freddy Haesebrouck, Frank Pasmans

**Affiliations:** 1 Department of Pathology, Bacteriology and Avian Diseases, Faculty of Veterinary Medicine, Ghent University, Merelbeke, Belgium; 2 Department of Basic Medical Sciences, Faculty of Medicine and Health Sciences, Ghent University, Ghent, Belgium; 3 Department of Pharmacology, Toxicology and Biochemistry, Faculty of Veterinary Medicine, Ghent University, Merelbeke, Belgium; University of California Riverside, United States of America

## Abstract

*Batrachochytrium dendrobatidis* (*Bd*) is the causative agent of chytridiomycosis, a fungal skin disease in amphibians and driver of worldwide amphibian declines.

We focussed on the early stages of infection by *Bd* in 3 amphibian species with a differential susceptibility to chytridiomycosis. Skin explants of *Alytes muletensis*, *Litoria caerulea* and *Xenopus leavis* were exposed to *Bd* in an Ussing chamber for 3 to 5 days. Early interactions of *Bd* with amphibian skin were observed using light microscopy and transmission electron microscopy. To validate the observations *in vitro*, comparison was made with skin from experimentally infected frogs. Additional *in vitro* experiments were performed to elucidate the process of intracellular colonization in *L. caerulea*.

Early interactions of *Bd* with amphibian skin are: attachment of zoospores to host skin, zoospore germination, germ tube development, penetration into skin cells, invasive growth in the host skin, resulting in the loss of host cell cytoplasm. Inoculation of *A. muletensis* and *L. caerulea* skin was followed within 24 h by endobiotic development, with sporangia located intracellularly in the skin. Evidence is provided of how intracellular colonization is established and how colonization by *Bd* proceeds to deeper skin layers. Older thalli develop rhizoid-like structures that spread to deeper skin layers, form a swelling inside the host cell to finally give rise to a new thallus.

In *X. laevis*, interaction of *Bd* with skin was limited to an epibiotic state, with sporangia developing upon the skin. Only the superficial epidermis was affected. Epidermal cells seemed to be used as a nutrient source without development of intracellular thalli. The *in vitro* data agreed with the results obtained after experimental infection of the studied frog species. These data suggest that the colonization strategy of *B. dendrobatidis* is host dependent, with the extent of colonization most likely determined by inherent characteristics of the host epidermis.

## Introduction

Chytridiomycosis is a lethal skin disease in amphibians caused by the fungal pathogen *Batrachochytrium dendrobatidis* (*Bd*). Causing widespread amphibian declines, this disease constitutes a major threat to amphibian biodiversity and conservation [1]–[4].

*Bd* colonizes the keratinized layers (stratum corneum) of amphibian skin or larval mouthparts [5]–[7]. Clinical infection is characterized by epidermal hyperplasia, hyperkeratosis and excessive shedding of the epidermis [5], [8]. Extensive colonization gives rise to a series of physiological effects such as disruption of the osmoregulatory function of the skin, leading to dehydration, electrolyte imbalance and death due to cardiac arrest [5], [9]–[13].

The lifecycle of *Bd* in culture and the pathology in skin from diseased animals are well documented [5], [8], [14]. Infection is established by zoospores, the motile flagellated stage of *Bd* [8], [14]. Zoospores display chemotactic responses in search of a suitable host to infect [15]. Upon colonization of the host epidermis, the zoospores encyst [8]. The flagellum is absorbed and a cell wall is formed [8]. Based on observations in infected *Litoria gracilenta*, an intracellular development of *Bd* was described by Berger et al. [8]. As such, the fungus proliferates within the epidermal cells and has its cycle tuned to the maturation of the epidermal cells [8]. Immature fungal bodies, termed thalli or sporangia are carried to the skin surface by differentiating epidermal cells [8]. Mature sporangia containing zoospores finally occur in the sloughing stratum corneum [8].

Early stages of infection have hitherto been poorly studied [16]. As such it is still not clear how host cell entry is achieved. In analogy with other pathogenic fungi e.g. *Candida albicans* [17] and dermatophytes [18], most probably a range of digestive enzymes capable of degrading skin components enable penetration of *Bd* into the host cells [16], [19], [20].

The main objective of this study was to find out how *Bd* infection is established. The early interaction between *Bd* and anuran skin was characterized using an *in vitro* infection model. Amphibian skin explants were inoculated with *Bd* and incubated in an Ussing chamber. To determine how *Bd* infection is established and to what extent infection strategies of *Bd* are host dependent, host-pathogen interactions were evaluated in 3 species with a differential susceptibility to *Bd*: the African Clawed Frog (*Xenopus laevis*), the Mallorcan Midwife Toad (*Alytes muletensis*) and the Green Tree Frog (*Litoria caerulea*). *X. laevis* generally does not show clinical signs associated with chytridiomycosis, nor have population declines due to chytridiomycosis been reported [21]. *A. muletensis* is a vulnerable European species restricted to Mallorca (Balearic Islands, Spain) [22]. Since *Bd* has been detected in reintroduced captive-bred populations this species is currently threatened by decline [22]–[23]. *L. caerulea* is a common Australasian species [22], but has proven to be highly susceptible to chytridiomycosis in the wild [22], [24] as well as under laboratory conditions [25], [26].

In a first experiment adhesion, invasion and the development of *Bd* in skin of *A. muletensis*, *L. caerulea* and *X. laevis* were studied during 3 to 5 consecutive days of *in vitro* infection using light microscopy (LM) and transmission electron microscopy (TEM). In parallel, *A. muletensis*, *L. caerulea* and *X. laevis* frogs were experimentally infected, to assess the validity of the observations

In a second experiment, skin of *L. caerulea* was exposed to *Bd* for 1, 2, 4, 8, 16 and 24 hours to further characterize the process of intracellular colonization. The morphology of the infecting fungal elements during invasion of the skin was followed by LM and TEM. The time-points of exposure found most critical for intracellular colonization were repeated in triplicate.

## Results

**Figure 1:**
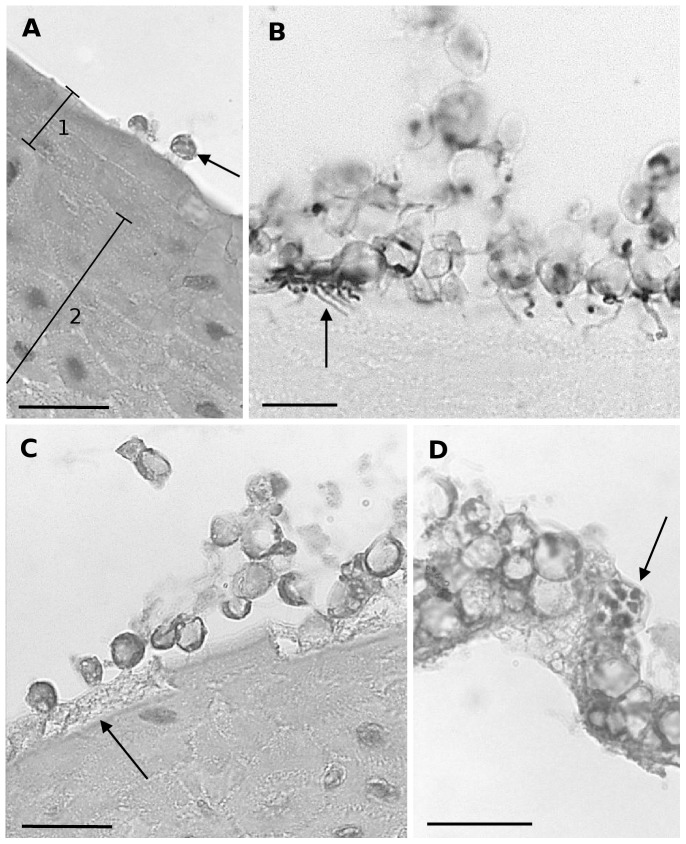
Light microscopical overview of the development of *Bd* in skin explants of *Xenopus laevis*. (**A**) adhesion of encysted zoospores (arrow) to the host epidermis at 1 dpi; (1) stratum corneum, (2) stratum spinosum; haematoxylin and eosin (HE) stain; scale bar = 20 µm; (**B**) at 1 dpi *Bd* germlings have developed germ tubes, that penetrate the stratum corneum and develop into a branched mesh work of rhizoids (arrow) in heavily infected epidermis; Gomori methenamine silver stain; scale bar = 10 µm; (**C**) at 2 dpi the infected host cells have lost their cytoplasm (arrow) subsequent to invasion by *Bd*, only the cell membrane remains; HE stain; scale bar = 20 µm; (**D**) at 4 dpi germlings have developed into mature zoosporangia (arrow), the upper layer of the stratum corneum is shed; HE stain; scale bar = 20 µm.

An overview of the early pathogenesis in *X. laevis* skin as observed by light microscopy is given in **Figure 1**. At 1 day post infection (dpi) numerous encysted zoospores had settled in clusters upon the epidermis or were situated in glandular pores (**Fig. 1A**). Zoospore cysts were spherical and had doubled in size (n = 30, (5)–6.1–(7.5) µm diameter) when compared to zoospores (n = 10, (2.0)–2.35–(3.5) µm diameter). From 1 dpi on, zoospore cysts germinated (termed germlings) and developed a short tubular structure of (0.5)–0.58–(0.86) µm diameter, further called germ tube (**Fig. 1B**). Germ tubes had elongated over the epidermal surface or had protruded into the cells of the stratum corneum. In heavily colonized cells the germ tubes growed into a profusely branched, fuzzy mesh work of rhizoids that spread out in the entire cell and was most clearly demonstrated by Gomori's methenamine silver stain (GMS) (**Fig. 1B**). At 2 dpi germlings had increased in size (n = 30, (5)–8.8–(13.2) µm diam.) and were developing into maturing zoosporangia. From 2 dpi on, invasion of the host cells of the stratum corneum resulted in loss of their cytoplasm and only their cell membrane persisted (**Fig. 1C**). Both at 3 and 4 dpi, the germlings upon the epidermal surface had matured into zoosporangia, containing zoospores. Several post-discharge zoosporangia were observed upon the epidermal skin surface. Sporangia were shed together with the affected upper layer of the stratum corneum (**Fig. 1D**).

**Figure 2:**
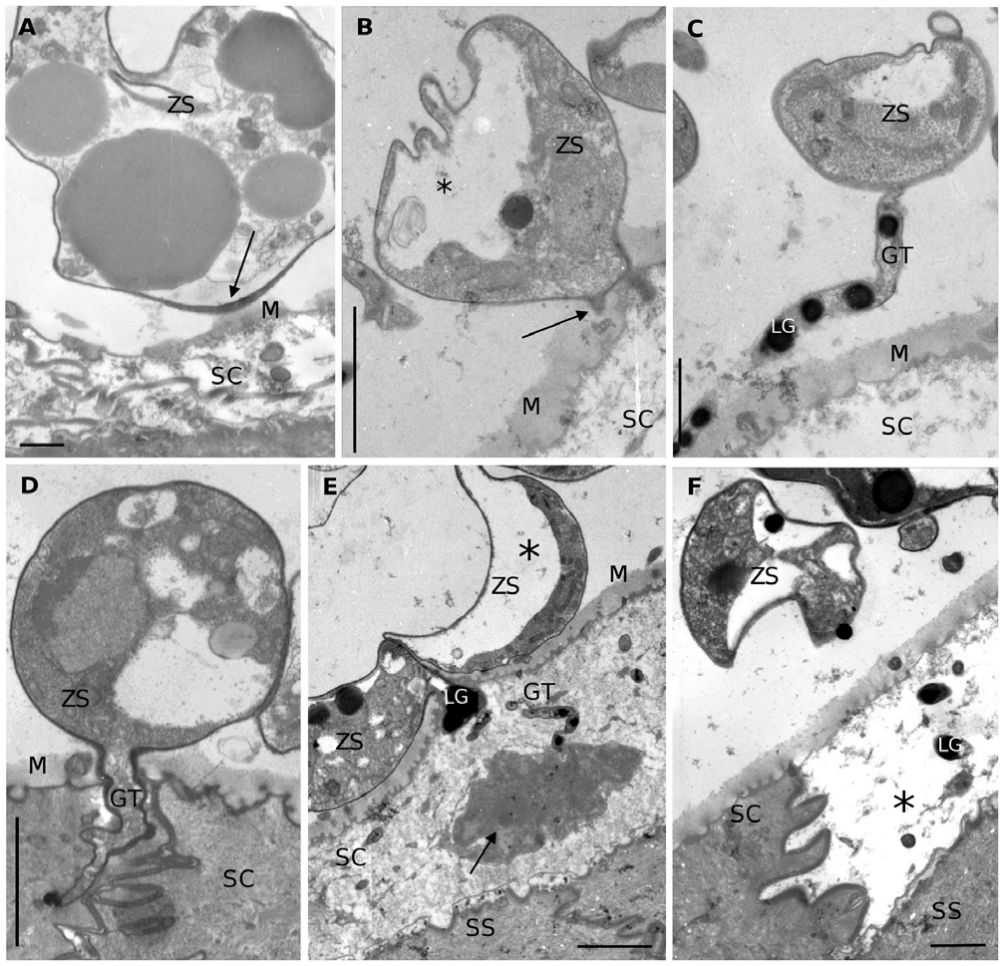
TEM overview of the development of *Bd* in skin explants of *Xenopus laevis*. (**A**) adhesion of an encysted zoospore (ZS) to the superficial mucus layer (M) on top of the stratum corneum (SC); at the site where adhesion occurs the cell wall of the encysted zoospore is remarkably thickened (arrow); scale bar = 500 nm; (**B**) initiation of germ tube development (arrow); note the polarisation of the cell cytoplasm (*); scale bar = 2 µm; (**C**) germ tube (GT) elongating upon the epidermis of *X. laevis*, with the presence of numerous lipid globules (LG) in the germ tube; scale bar = 1 µm; (**D**) a growing germ tube protruding the stratum corneum; scale bar = 2 µm; (**E**) invasion of a host cell resulting in the loss of cell cytoplasm; remnants of the host cell cytoplasm (arrow) are seen at the tip of a protruded germ tube; note the presence of a collapsed sporangium (ZS) due to cell polarisation (*); (SS): stratum spinosum; scale bar = 2 µm; (**F**) infected epidermal cell with digested cell content (*) alternated by an uninfected normal epidermal cell; note the presence of lipid globules in the infected host cell; scale bar = 1 µm.

TEM provided more detailed information on ultrastructural changes (**Fig. 2**). At 1 dpi, encysted zoospores were attached to a thin residual superficial mucus layer on top of the stratum corneum and adhesion to this layer was characterized by a conspicuous thickening of the fungal cell wall (a 3 to 6 fold increase, from 0.05–0.1 µm to 0.2–0.3 µm) (**Fig. 2A**). The initiation of a germ tube, as shown in **Figure 2B**, started as a pointed outgrowth of the thickened cell wall. Cross-sections of a germ tube by TEM show prominent osmiophilic rounded structures inside the germ tube (**Fig. 2C**). These structures without a membrane and of variable size resembled the lipid globules in the zoospores of *Bd*. Analogous structures were seen in the cytoplasm of the affected keratinocytes (**Figs. 2E,F**). No mitochondria or nuclei could be discerned in these germ tubes. **Figure 2D** shows a growing germ tube that had protruded in the stratum corneum. The affected epidermal cells seemed to have partially or completely lost their cytoplasm and only their cell membrane persisted (**Figs. 2E,F**). Remnants of the host cell cytoplasm were observed at the tip of an invading germ tube (**Fig. 2E**). In un-inoculated skin samples, incubated during 5 days under the same conditions as described above, the stratum corneum was still intact and no altered keratinocytes were observed (**Fig. S1**).

Another striking feature seen from 1 dpi onwards was the presence of collapsed sporangia. This was observed in histological and TEM preparations (**Fig. 2E**). A polarisation of the sporangial cytoplasm was observed. The cytoplasm was concentrated at one side of the sporangium, lined by an empty space most probably to be considered as a vacuole.

**Figure 3:**
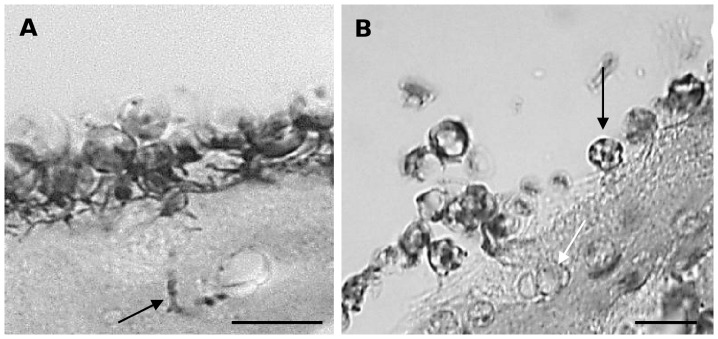
Light microscopical overview of the development of *Bd* in skin explants of *Alytes muletensis* and *Litoria caerulea*. (**A**) at 1 day post infection (dpi) germlings have developed germ tubes (arrow) that invade the epidermis of *A. muletensis*; Gomori methenamine silver (GMS) stain; scale bar = 10 µm; (**B**) at 1 dpi both *Bd* germlings (black arrow) attached upon the epidermal surface as intracellular chytrid thalli (white arrow) in the stratum corneum of *L. caerulea* are observed; haematoxylin and eosin stain; scale bar = 10 µm.

**Figure 4:**
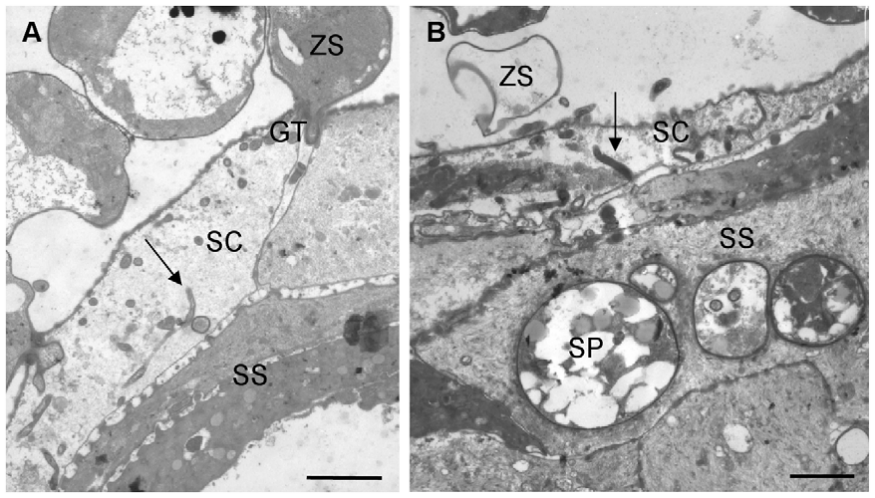
TEM overview of the development of *Bd* in skin explants of *Alytes muletesis and Litoria caerulea*. (**A**) infected epidermis of *A. mulentensis* at 1 dpi, with loss of the host cell cytoplasma and the presence of germ tube fragments inside the infected cell in cross and longitudinal section (arrow); scale bar = 2 µm; (**B**) infected epidermis of *L. caerulea* at 2 dpi showing colonization of the stratum corneum, loss of the host cell cytoplasm and the presence of germ tube fragments (arrow); intracellular chytrid sporangia are observed in the stratum spinosum; scale bar = 2 µm; GT; germ tube, SC: stratum corneum, SP: sporangium, SS: stratum corneum, ZS: encysted zoospore.

**Figure 3** illustrates the development of *Bd* in skin explants of *A. muletensis* and *L. caerulea*. Compared to *X. laevis*, a similar initial infection process was seen in *A. muletensis* and *L caerulea*. Likewise, zoospore cysts adhered to the stratum corneum, host cells were invaded by germ tubes that developed into rhizoidal axes spreading out in the entire cell (**Figs. 3A,B**). Invasion of the keratinocytes by germ tubes and loss of the cellular cytoplasm was most obvious by TEM (**Figs. 4A,B**). From 2 dpi on, maturing sporangia were observed upon the infected skin surface. However, the development of *Bd* in *A. muletensis* and *L. caerulea* was clearly distinct in the respect that besides superficial colonization, intracellular chytrid thalli were observed in superficial and deeper layers of the epidermis. Within 24 hours after inoculation, marked intracellular colonization was seen in *L. caerulea* by LM (F**ig. 3B**) and TEM (**Fig. 4B**) and occasional intracellular colonization in *A. muletensis*.

To support the validity of the observations made *in vitro*, *A. muletensis, L. caerulea* and *X. laevis* frogs were infected *in vivo*. During the course of the infection trial no clinical signs were observed in *A. muletensis* (n = 3). In 1 out of 3 *L. caerulea* excessive shedding of skin and erythema of the hind limbs occurred. In all *X. laevis* frogs (n = 3) only excessive shedding of skin was observed. At 12 days post infection, all *A. muletensis* and *L. caerulea* frogs were infected, with mean genomic equivalents of *Bd* ± standard error detected by qPCR of 517 ± 636 for *A. muletensis* (n = 3) and 350 ± 589 for *L. caerulea* (n = 3). All *X. laevis* frogs tested negative (n = 3).

**Figure 5:**
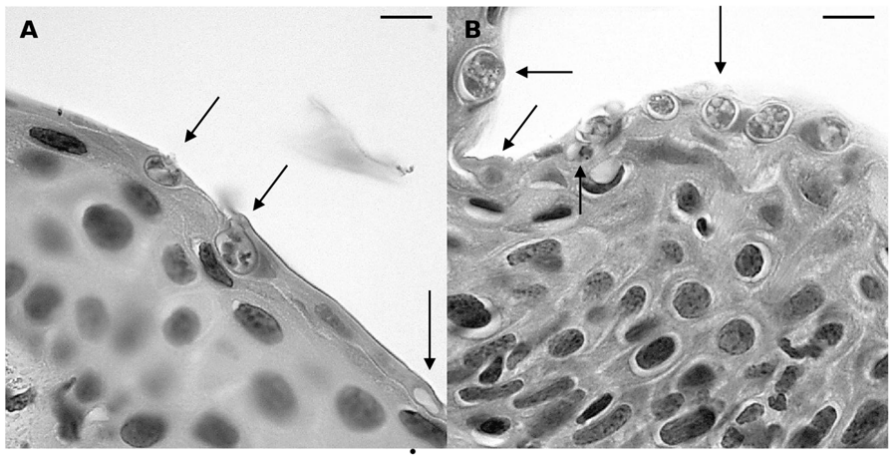
Skin sections of *Alytes muletensis* and *Litoria caerulea* experimentally infected with *Bd* at 14 days post infection. Exclusively intracellular chytrid thalli (arrows) are observed by light microscopy in the stratum corneum of *A. muletensis* (**A**) and *L.caerulea* (**B**); haematoxylin and eosin stain; scale bar = 10 µm.

In skin samples taken at 14 days after exposure, the epidermis of experimentally infected *X. laevis* frogs was still intact. No adhering zoospores nor sporangia could be observed. In contrast, in all infected *A. muletensis* (n = 3) (**Fig. 5A** **, LM**) and *L. caerulea* frogs (n = 3) the stratum corneum was colonized with intracellular sporangia. Germlings or developing sporangia adhering to the epidermis were not observed. Colonization was more abundant in *L. caerulea*. One out of 3 infected *L. caerulea* frogs carried a high infection load (1020 GE), was colonized to broad extent (**Fig. 5B** **, LM**) but did not show any clinical signs. The other *L. caerulea* individuals were infected to the same and lesser extent (9 and 11 GE), with only one individual presenting clinical signs.

**Figure 6:**
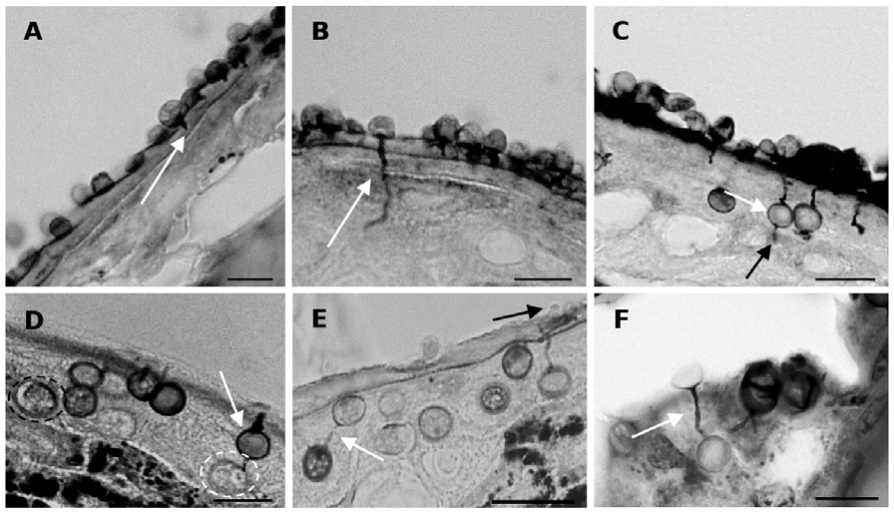
Intracellular colonization of *Litoria caerulea* skin by *Bd*. (**A–E**): *in vitro*, (**F**): *in vivo*. (**A**) invasion of the stratum corneum by a germ tube (white arrow) at 2 hour post infection (hpi); (**B**) strong elongation of the germ tube (white arrow) into the stratum spinosum at 8 hpi; (**C**) development of intracellular chytrid thalli (white arrow) at the end of a germ tube at 24 hpi; rhizoid-like structures (black arrow) arise from newly developed chytrid thalli; (**D**) development of a new chytrid thallus at 24 hpi; a swelling is formed at the end of a rhizoid-like structure, a thin cell wall is formed and the cell content of the mother thallus (white arrow) is transferred into the new daughter thallus (white circle); a new thallus in a later developmental stage (black circle); (**E**) thalli connected by a rhizoid-like structure (white arrow); remnants of a germling, after having injected its cell content into a new intracellular thallus (black arrow); (**F**) mother thallus connected to a newly formed daughter thallus by a rhizoid-like structure (white arrow) at 14 days post infection. Gomori methenamine silver stain, scale bar = 10 µm.

A more detailed study of the invasion process in *L. caerulea* showed that at earliest, frog skin was invaded by germ tubes 2 hours after exposure to *Bd* (**Fig. 6A,B**). Eight, 16 and 24 hours of exposure to *Bd* were defined as most critical time-points to study intracellular colonization and were repeated in triplicate during additional *in vitro* assays. Chytrid thalli developing intracellularly were observed at 16 to 24 hours after exposure. In one out of the 3 repeats, intracellular colonization occurred 8 hours after exposure. In this experiment the stratum corneum had already detached from the stratum spinosum, probably rendering the epidermis more accessible.

GMS staining showed that both superficially and deeper localized intracellular chytrid thalli were often connected to a tubular rhizoid-like structure, stretching out to the deeper layers of the epidermis or either to the epidermal surface (**Figs. 6C,D**). In rare cases, remnants of empty zoospore cysts were found at the skin surface (**Fig. 6E**). On several occasions, both *in vitro* (**Fig. 6E** **)** and *in vivo* (**Fig. 6F**) intracellular chytrid thalli apparently connected by a rhizoid-like structure were noticed. As such, older thalli were connected to newly formed thalli. **Figure 6D** shows a clearly stained older thallus giving rise to a new thallus, outlined by a faintly stained thin cell wall.

## Discussion

The present results provide a missing link in the infection process of *Bd*. Until now established *Bd* infections had been described with zoospore development occurring in a zoosporangium inside the host cell and the intracellular zoosporangium forming discharge papillae through which zoospores exit [8]. Our results provide a novel insight into the early interaction of *Bd* zoospores with amphibian skin.

The early pathogenesis consists in the first place of an epibiotic development, upon the host skin and was observed in the 3 species studied. Zoospores matured into thick-walled cysts on the host epidermis and were clustered in foci of infection. Subsequently, invasion of amphibian skin was established by germ tube development. A tubular extension or germ tube arised from the zoospore cysts and penetrated into the epidermal host cells. GMS stained sections showed most clearly that in heavily infected cells germ tubes grew into an irregularly branched mesh work of rhizoids. Histological sections and TEM images strongly suggest an extracellular digestion of the host cytoplasm, followed by an uptake by the germ tubes. A similar effect has been described by Berger et al. [8], who observed dissolution of cellular cytoplasm in infected epidermis of *L. gracilenta*. However, this was not associated with the presence of germ tubes.

*In vitro*, *X. laevis* skin does become infected but the development of *Bd* in *X. laevis* skin is apparently limited to an ‘epibiotic’ stage, with epidermal cells solely being used as nutrient source for the growing sporangium upon the epidermis. The typical histological picture of chytridiomycosis with chytrid sporangia developing intracellularly was not observed. Upon examination of stained skin sections from *in vivo* infected *X. laevis* frogs, 14 days after exposure to *Bd*, the skin was still intact and no colonization was observed.

*X. laevis* is considered tolerant to clinical chytridiomycosis as defined by Schneider & Ayers [27], i.e. this species can be colonized by *Bd* but is able to limit the impact of *Bd* on its health and to maintain a low-level infection [21], [28]. Unfortunately, reports of chytrid infections in *X. laevis* rely merely on PCR-detection [21], [29], [30] and there is no conclusive histological evidence of how chytrid infections manifest in this species under natural conditions.

Recently, Ramsey et al. [31] found that the level of infection in *X. laevis* is likely to be determined by both innate and adaptive components of the immune system. As such antimicrobial peptides [32], [33] and antifungal metabolites [34] provide a non-specific protection to potential pathogens, while antibodies in skin secretions of previously exposed frogs provide specific anti-*Bd* protection. A combined action of these defenses is likely to limit colonization of *X. laevis* by *Bd* to mild and non-lethal infections. To which extent epibiosis occurs in other chytrid tolerant species remains to be determined. For example, though the American Bullfrog (*Lithobathes catesbeianus*) is considered a notorious carrier of *Bd*, there is solid evidence of *Bd* developing intracellularly in the skin of this species [35], [36].

In *A. muletensis* and *L. caerulea* epibiotic development was followed by extensive intracellular colonization of the stratum corneum. Intracellular growth of chytrid thalli was established within 24 hours in *L. caerulea* and in the later stages of infection in *A. muletensis*. Additional infection assays in *L. caerulea* confirm that colonization propagates to the deeper skin layers within 24 hours, at earliest at 16 hours after exposure to *Bd*. Both in the wild [22]–[24] as in experimental infection trials [25], [26], [37] *A. muletensis* and *L. caerulea* can be severely colonized by *Bd*.

**Figure 7:**
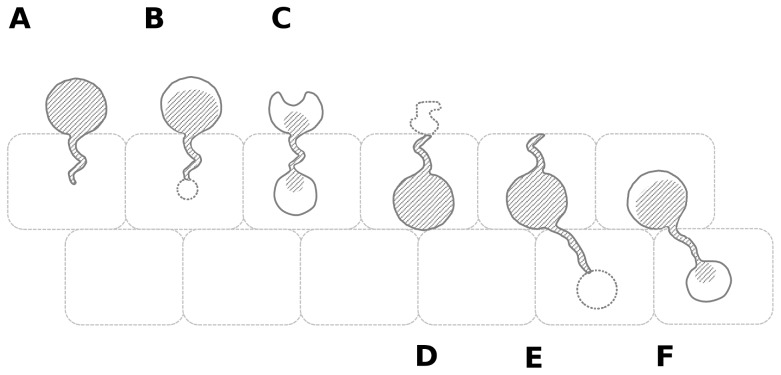
Schematic summary of the intracellular colonization process by *Bd* in amphibian skin. (**A**) Germination of a zoospore cyst or germling is followed by the development of a germ tube that invades an epidermal cell; (**B**) at the end of the germ tube a swelling is formed, that gives rise to a new thallus; (**C**) cell contents of the germling migrate into the newly formed thallus; (**D**) the emptied germling evanesces; (**E**) the new intracellular thallus forms a rhizoid-like structure that extends to a deeper epidermal layer and develops a swelling at its end; (**F**) a new intracellular thallus is formed.

Especially GMS staining proved its usefulness in visualising fungal cell walls and revealing structures that were overlooked using HE staining. In *A. muletensis* and *L. caerulea* intracellular chytrid thalli with rhizoid-like structures stretching out either to the epidermal surface or to the deeper layers of the epidermis were observed. On several occasions, both *in vitro* and *in vivo*, intracellular chytrid thalli apparently connected by a rhizoid-like structure were noticed. These observations provide consistent evidence of how intracellular colonization is established, as summarized in **Figure 7**. Together with the presence of germ tubes these data confirm the hypothesis of an endobiotic development of *Bd* as formulated by Longcore [14]. During endobiotic development zoospores encyst upon the host cell and inject their nucleus and cytoplasm into the host cell via a germ tube. The germ tube forms a swelling inside the cell and enlarges. Finally, the contents undergo mitosis, zoospores are formed and are released into the environment through discharge papillae [14]. Analogous invasion mechanisms are seen in chytrids parasitizing plants and algae e.g. *Entophlyctis* spp. [38], [39].

In addition, our observations indicate how colonization by *Bd* proceeds to deeper skin layers. It seems that older mother thalli develop rhizoid-like structures that spread to the deeper skin layers and form a swelling inside the cell. This swelling enlarges and gives rise to a new daughter thallus.

Genetic material of *Bd* was probably injected into epidermal cells in order to establish intracellular sporangia. As we did not observe any mitochondria or nuclei in the germ tubes additional observations are desirable. However, migration of lipid globules through these germ tubes into the host cell was seen on several occasions. Most probably the lipids function as a source of concentrated energy for the zoospores and as an energy source for the young thallus while it grows into an epidermal cell (pers. comm. J. Longcore).

Host induced morphological variation is peculiar in many chytrids [40], i.e. they exhibit morphological differences between their parasitic and saprophytic state and are able to change from an endobiotic growth to an epibiotic growth depending on nutrients and the substrate [38], [40]. In this perspective, our data suggest that the colonization strategy of *Bd* is host dependent. The extent of *Bd* invasion clearly differed between the 3 host species used: from near absence in *X. laevis*, to moderate in *A. muletensis* and high in *L. caerulea*. Moreover, the ability of *Bd* to enter amphibian skin and to spread in the skin, or its invasiveness, appears to coincide with the susceptibility of the studied species to chytridiomycosis, e.g. low in *X. laevis* [21], [28], [41], moderate in *A. muletensis* [23], [37] and high in *L. caerulea* [24]–[26]).

Why colonization is limited to epibiosis in *X. laevis* and what makes *A. muletensis* and *L. caerulea* more ‘receptive’ to *Bd* infection remains speculative. However, the influence of certain factors on the outcome of the experiments was minimized. *In vitro* colonization experiments were carried out under the same conditions. Prior to the isolation of skin explants *A. mulentensis*, *L. caerulea* and *X. laevis* frogs were washed to facilitate the handling of skin tissue and to reduce the risk of bacterial contamination. By washing, skin mucus and skin secretions were also partially removed. Especially *X. laevis* skin is covered with a prominent mucus layer and skin mucus in itself can be considered as a mechanical barrier and an obstacle for colonization. In addition, skin secretions containing e.g. antimicrobial peptides, antifungal metabolites, are thought to provide protection against chytridiomycosis. However, since the activity of residual skin secretions in skin explants is not yet studied, one must be cautious in assuming a reduced defensive action.

Consequently, we hypothesize that the degree of invasiveness of *Bd* and amphibian susceptibility to chytridiomycosis is determined by inherent characteristics of the host skin. However, more observations are required to draw definite conclusions about species susceptibility and pathogenesis patterns. The challenge ahead will be to identify which factors mediate these variations in the pathogenesis of *Bd* infections.

## Materials and Methods

### Experimental animals

Postmetamorphic wild type *X. laevis* were purchased from the European *Xenopus* Resources centre (Portsmouth, UK) and adult outbred *X. laevis* from *Xenopus* Express (Le Bourg, France). Subadult *A. mulentesis* and *L. caerulea* were captive bred. Upon arrival and before the start-up of all experiments skin swabs from all animals were examined for the presence of *Bd* by the quantitative PCR (qPCR) of Boyle et al. [42].

### *Bd* strains and culture conditions

Inoculations were carried out with the virulent *Bd* strain IA042, a representative of the *Bd* global panzootic lineage [43], isolated from a dead *Alytes obstetricans* [44]. Cultures were maintained on tryptone/gelatine hydrolysate/lactose (TGhL) broth in 25 cm^2^ cell culture flasks at 20°C for 5 days. Two ml of a 5 days old broth culture were inoculated on TGhL agar and incubated for 5 to 7 days at 20°C. Zoospores were harvested by flooding the agar plates with 2 ml of distilled water and were immediately counted in lugol with a haemocytometer.

### Isolation *in vitro* culture and infection of anuran skin

For a detailed study of the early interaction between zoospores and host epidermis, full- thickness epidermal (FTE) explants of *A. mulentensis*, *L. caerulea* and *X. laevis* were experimentally infected in an Ussing chamber based model. Isolation and treatment of FTE explants, *in vitro* culture and infection procedures have been described in detail by Van Rooij et al. [45].

Early interactions of *Bd* zoospores with amphibian epidermis and the *in situ* development of zoospores to sporangium were observed during 5 consecutive days in *X. laevis* and 3 days in *A. muletensis* and *L. caerulea*. Immediately after euthanasia with intracoelomically-injected T 61® (Intervet, Mechelen, Belgium) frogs are washed according to the protocol of Nishikawa et al. [46] to facilitate the handling of the tissue and to reduce the risk of contamination. Briefly, frogs were washed in plastic containers containing respectively 70% ethanol, Leibovitz L-15 medium 70% (3 times; Gibco, Life technologies Europe, Gent, Belgium), Ca^2+^/Mg^2+^-free Barth's solution (CMFB), 1.25 mM ethylenediaminetetraacetic acid (EDTA; Sigma, St. Louis, MO, USA) in CMFB for 5 min and 70% L15 medium (twice) at 4°C. FTE explants (10×25 mm for *A. muletensis* and 20×25 mm for *L. caerulea* and *X. laevis*) were excised and were mounted in an Ussing chamber (exposed surface area of 0.28 cm^2^ for *A. muletensis* and 1.07 cm^2^ for *L. caerulea* and *X. laevis*). From each donor animal a skin sample was tested for the presence of *Bd* by qPCR [42].

Explants were apically exposed to 7 ml inoculum (2.8×10^7^ zoospores/ml distilled water). In the course of the subsequent 3 to 5-days incubation period at 20°C, skin samples were removed at 1 to 3–5 days post infection (dpi) and the exposed skin surface area was excised and processed for histology and transmission electron microscopy (TEM).

Additional experiments in skin of *L. caerulea* were performed under the same conditions as described above. In short, FTE skin explants were mounted in an Ussing chamber with an exposed surface area of 1.07 cm^2^. Explants were exposed to an inoculum of 2.8×10^7^ zoospores/ml distilled water. In the course of a 24 hours incubation period at 20°C, skin samples were removed after 1, 2, 4, 8, 16 and 24 hours of exposure. The experiment was then repeated in triplicate, with sampling after 8, 16 and 24 hours of exposure.

For light microscopic studies, skin samples were fixed in 10% neutral buffered formalin and embedded in paraffin. Five µm sections were stained with haematoxylin and eosin (HE) and Gomori methenamine silver (GMS). For TEM, skin samples were fixed in 4% formaldehyde containing 1% CaCl_2_ (w/v) in 0.121 M Na-cacodylate adjusted to pH 7. The samples were washed and postfixed in 1% OsO_4_ (w/v). Subsequently the skin samples were dehydrated through a graded series of alcohol and embedded in LX-112 resin (Ladd Research Industries, Burlington, Vermont, USA). Semi-thin sections (2 µm) were cut and stained with toluidin blue to select regions for ultrathin sectioning (90 nm) with an ultratome (Ultracut E; Reichert-Jung, Nussloch, Germany). The ultrathin sections (90 nm) were stained with uranyl acetate and lead citrate solutions and examined under a JEOL EX II transmission electron microscope (JEOL Ltd, Zaventem, Belgium) at 80 kV. Measurements of all structures are given as (Min.)-Av.-(Max.), with Min. = minimum value for the measured collection of structures (n), Av. = average value and Max. = maximum value.

### *In vivo* infection of *A. muletensis*, *L. caerulea* and *X. laevis*

To assess the validity of the *in vitro* results, *A. mulentensis*, *L. caerulea* and *X. laevis* frogs were experimentally infected. All animal experiments were approved by the Ethical Committee of the Faculty of Veterinary Medicine, Ghent University, Belgium (EC2008/120, EC2010/98). Experiments were performed following all necessary ethical and biosecurity standards.

For inoculation, respectively three subadult *A. mulentensis*, *L. caerulea* and *X. laevis* frogs were individually housed for 24 hours in plastic containers (18×13×4 cm) containing moistened paper tissue, terracotta flower-pots as shelter and a petri-dish filled with dechlorinated tap water for bathing. Frogs were inoculated by topical application of 2×10^6^ zoospores/100 µl distilled water (*Bd* strain IA042). Twenty-four hours after exposure all frogs were transferred to fresh containers. *X. laevis* frogs were housed in plastic containers (32×17×21 cm), containing dechlorinated tap water (water depth 10 cm) at 20°C. Terracotta flower-pots were provided as shelter. Frogs were fed twice weekly with trout pellets (Skretting, Cheshire, UK). Water was changed three times weekly. *A. muletensis* and *L. caerulea* frogs were housed in plastic containers (18×13×4 cm), with moistened paper tissue, terracotta flower-pots as shelter and a petri-dish filled with dechlorinated tap water for bathing. Containers were regularly sprayed with dechlorinated tap water to maintain humidity. Tissue and bathing water was changed three times weekly. Ambient temperature varied between 19 and 20°C. *A. muletensis* and *L. cearulea* frogs were fed calcium powdered crickets, fruit flies (*Drosophila melanogaster*) or buffalo worms (*Alphitobius laevigatus*) *ad libitum*. The photoperiod of the experimental animal facilities followed natural ambient conditions (12–15 h light). At day 12 post inoculation all animals were sampled by passing a sterile synthetic swab (160 C, Copan Italia S.p.A., Brescia, Italy) along the pelvic region 10 times, fore- and hind limbs 5 times. Gloves were changed between handling of each animal. Swabs were examined for the presence of *B. dendrobatidis* by qPCR [42]. At day 14 post inoculation all animals were sacrificed by intracoelomically-injected T 61® (Intervet, Mechelen, Belgium). Skin samples were collected from the pelvic region, fixed in10% neutral buffered formalin, processed for histology and stained with HE and GMS.

## Supporting Information

Figure S1 **TEM image of negative control skin explant of** ***Xenopus laevis*** **.** Negative control sample, incubated with distilled water during 5 days under the same conditions as skin explants exposed to *Bd*. zoospores; all cell layers are still intact; scale bar = 5 µm.
(TIF)
