# Supplementary figures and images for: Germ Tube Mediated Invasion of Batrachochytrium dendrobatidis in Amphibian Skin Is Host Dependent
Source: PLoS One. 2012 Jul 20;7(7):e41481. doi: 10.1371/journal.pone.0041481 (PMC3401113; doi:10.1371/journal.pone.0041481)

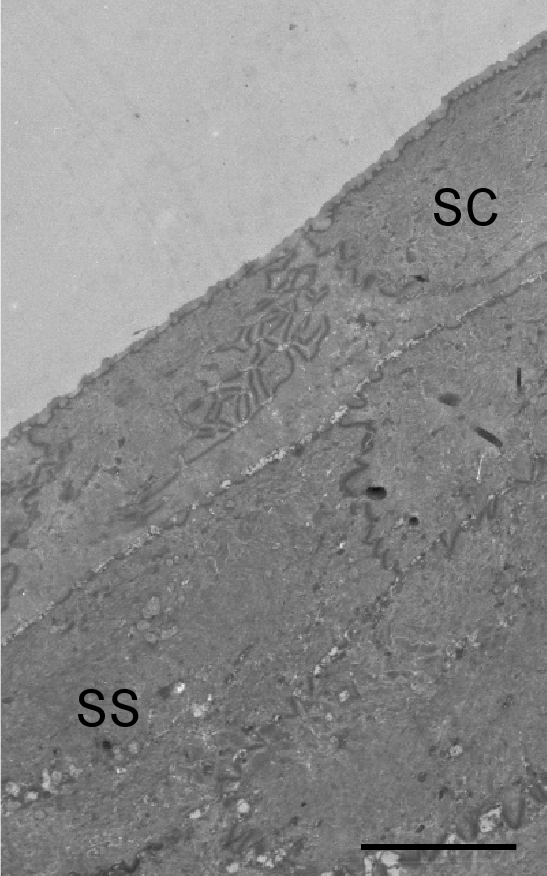

Supplement: Figure S1 — TEM image of negative control skin explant of Xenopus laevis . Negative control sample, incubated with distilled water during 5 days under the same conditions as skin explants exposed to Bd. zoospores; all cell layers are still intact; scale bar = 5 µm. (TIF) [file pone.0041481.s001.tif]
